# Clinical impact of cerebral near-infrared reflectance spectroscopy on the management of severe traumatic brain injury

**DOI:** 10.1016/j.cjtee.2025.05.011

**Published:** 2026-05-29

**Authors:** Panu Boontoterm, Siraruj Sakoolnamarka, Karanarak Urasyanandana, Pusit Fuengfoo

**Affiliations:** a Neurological Surgery Unit, Department of Surgery, Phramongkutklao Hospital, Bangkok, Thailand; b Department of Surgery, Phramongkutklao Hospital, Bangkok, Thailand

**Keywords:** Brain injuries, Traumatic, Cerebral near-infrared reflectance spectroscopy, Regional cerebral oxygen saturation, Cerebral desaturation, Neurosurgical intensive care unit

## Abstract

**Purpose:**

Severe traumatic brain injury (sTBI) is associated with cerebral hypoxia and hemodynamic instability, contributing to secondary brain injury and poor neurological outcomes. This prospective pilot study evaluated whether cerebral near-infrared reflectance spectroscopy (NIRS)-guided monitoring with interventions to maintain the regional cerebral oxygen saturation (rSO_2_) ≥55% improves outcomes in patients with sTBI.

**Methods:**

Eighty adult patients with sTBI requiring neurosurgical intervention were prospectively randomized into an intervention group (*n* = 40) receiving continuous cerebral NIRS monitoring with protocol-guided optimization of rSO_2_, or a control group (*n* = 40) receiving standard neurocritical care. Eligible patients were aged >20 years with Glasgow coma scale scores <8. Baseline demographic and clinical characteristics were comparable between groups. Interventions included optimization of head position, ventilation, mean arterial pressure (MAP), cardiac index, oxygen supplementation, and transfusion when indicated. Primary outcomes included mortality, intensive care unit and hospital length of stay, and complications. Secondary outcomes included neurological recovery assessed using the modified Rankin scale and Glasgow outcome scale–extended at 1-year follow-up. Statistical analyses were performed using Chi-square, Fisher's exact, *t*-test, Mann-Whitney *U* tests, etc.

**Results:**

Hospital mortality was lower in the intervention group than in the control group (45.0% *vs.* 47.5%, *p* = 0.043). Favorable neurological outcomes (mRS 0 – 3) at 1 year were significantly more frequent in the intervention group (25.0% *vs.* 5.0%, *P* = 0.006). No significant differences were observed in ICU length of stay, ventilator-free days, vasopressor-free days, or major complications. Low cardiac index ≤2.0 L/(min·m^2^) and MAP ≤65 mmHg were independent predictors of mortality. Mean rSO_2_ positively correlated with intensive care unit length of stay (*r* = 0.459, *p* < 0.001).

**Conclusion:**

NIRS-guided cerebral oxygenation monitoring is feasible and associated with improved long-term neurological outcomes in sTBI patients. Maintaining adequate MAP and cardiac index may be critical for optimizing cerebral oxygenation and reducing mortality risk.

**Clinical trial registration:**

ClinicalTrials.gov (NCT06306950)

## 1 Introduction

Near-infrared reflectance spectroscopy (NIRS) provides real-time data on cerebral and somatic tissue oxygenation, offering insights into the balance between oxygen supply and demand. This technique has become an essential tool for identifying the causes of impaired regional brain oxygenation and perfusion.^1^ NIRS enables continuous monitoring of oxygenation and has been evaluated across various patient populations, including adults and children, and in multiple surgical specialties such as cardiac, neurosurgery, vascular surgery, and cardiopulmonary procedures. It has also been applied in severe traumatic brain injury (sTBI) cases. NIRS is capable of assessing bifrontal cortical regions and regional cerebral oxygen saturation (rSO_2_). Studies have shown correlations between low rSO_2_ values and adverse outcomes in sTBI, including cognitive dysfunction, prolonged stays in the neurosurgical-intensive care unit (ICU), and extended hospitalizations,^2^ as well as perioperative stroke.^3^

In this study, we utilized cerebral oximetry monitoring,^4^ recognizing that cerebral deoxygenation (rSO_2_ <55%) is associated with multiple systemic complications, such as renal failure, poor overall well-being,^5^ prolonged mechanical ventilation,^6^ altitude sickness,^7^, ^8^, ^9^ impaired cortical function,^10^ hypoxia,^11^ and low cardiac index (CI) syndrome.^12^ Continuous monitoring has reinforced these correlations, suggesting that interventions to optimize cerebral oxygenation could benefit systemic perfusion. Many intraoperative measures to optimize rSO_2_, such as adjusting PaCO_2_, blood flow rates, and arterial blood pressure, also influence systemic perfusion. Thus, we hypothesized that using the brain as an index organ and implementing strategies to maintain rSO_2_ above 55% would result in systemic benefits, including improved global tissue perfusion and better clinical outcomes.^13^, ^14^, ^15^, ^16^ Based on this, we postulated that NIRS monitoring, combined with a targeted treatment protocol to optimize rSO_2_ would improve outcomes in sTBI patients. NIRS monitoring of rSO_2_ in these patients enables interventions to maintain rSO_2_ levels above 55%, thereby improving both perioperative and overall outcomes.^17^

## 2 Methods

### 2.1 Patient enrollment

This study was designed as a prospective, randomized, pilot exploratory trial to evaluate the feasibility, safety, and preliminary clinical impact of cerebral oxygenation optimization using NIRS in patients with sTBI. Because limited prior evidence and insufficient preliminary data were available regarding the expected effect size of NIRS-guided interventions in this patient population, the sample size estimation was exploratory in nature. Therefore, the study should be interpreted primarily as a pilot investigation intended to assess the practicality of the monitoring protocol, generate preliminary efficacy data, and provide foundational information for the design and power calculation of future large-scale multicenter randomized controlled trials.

The trial was conducted at a tertiary-care neurotrauma center between April 1, 2021 and January 31, 2023. All consecutive patients during the study period presenting with sTBI who were scheduled for neurosurgical intervention at our hospital were assessed for eligibility. Following initial stabilization, written informed consent was obtained from each patient's legal guardian or next of kin within 4 h of hospital admission and prior to enrollment.

The inclusion criteria were (1) age >20 years; (2) Glasgow coma scale (GCS) score <8; and (3) diagnosis of sTBI requiring neurosurgical intervention. The exclusion criteria were: (1) pregnancy; (2) forehead infection; (3) status epilepticus; (4) history of drug addiction; (5) diffuse brain injury classified as Marshall grade III – VI; or (6) presence of metabolic complications.

### 2.2 Patient grouping

Patients were randomized in a 1:1 ratio to either the NIRS-monitored intervention group or the control group receiving standard care. Randomization was achieved using a computer-generated sequence with treatment allocations sealed in opaque, consecutively numbered envelopes. The envelopes were opened upon the patient arrival in the emergency department.

To maintain blinding, the NIRS monitor screen was visible only to the ICU clinical team implementing the interventions; the monitor was electronically blinded to data collectors and outcome assessors to reduce observation bias; and the study group identifiers were omitted from patient medical records to preserve allocation concealment throughout the trial.

A total of 135 patients diagnosed with sTBI were assessed, and 80 were finally enrolled in the study and randomized into either the NIRS intervention group (intervention group, *n* = 40) or the control group (*n* = 40) (Fig. 1). All randomized participants were included in the final analysis based on the intention-to-treat principle. There were no exclusions after randomization, and no patients were lost to follow-up during the study period.

**Fig. 1:**
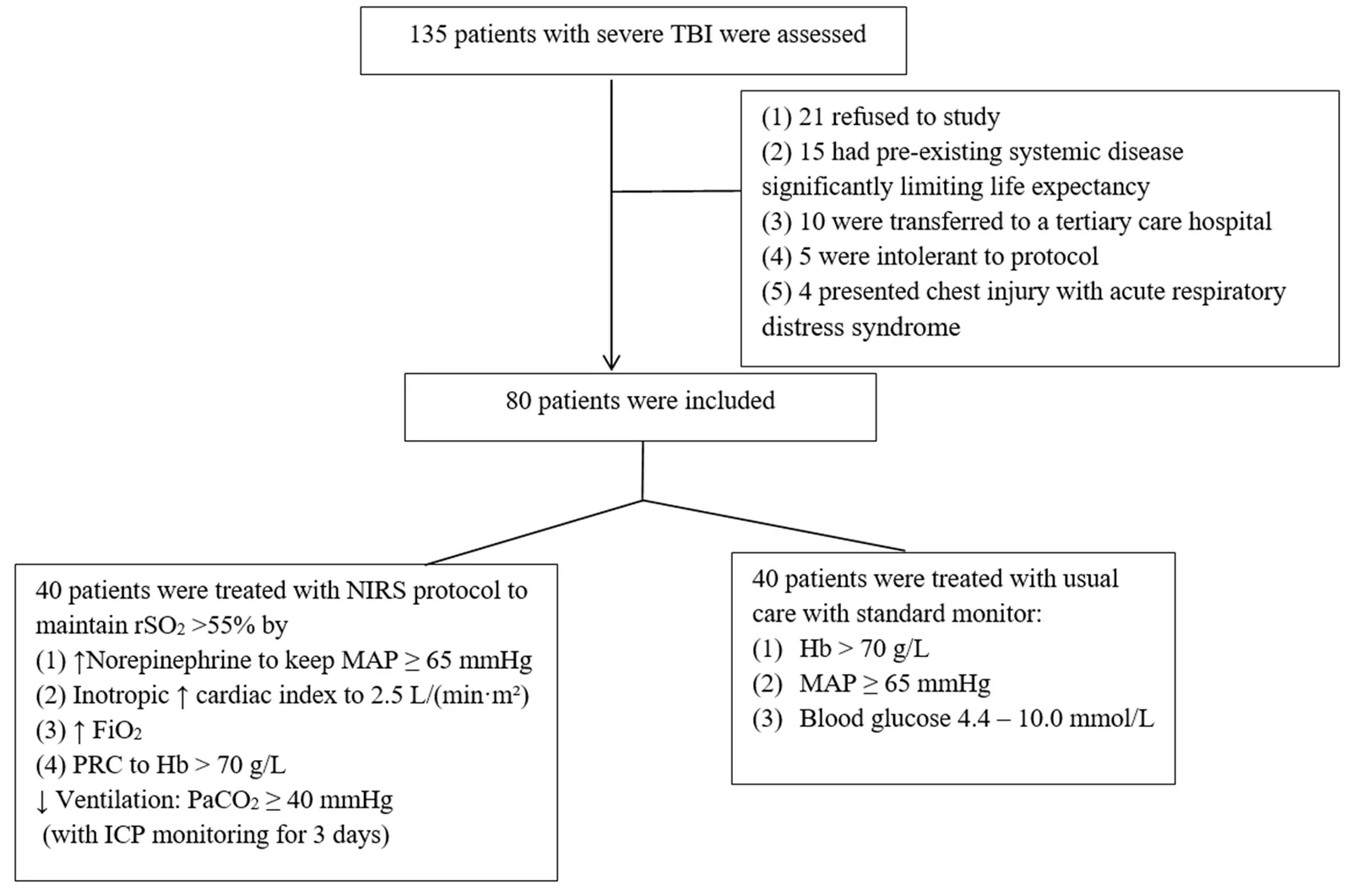
Flow diagram of patient enrollment, treatment allocation, and analysis. All the 80 patients were included in the primary outcome analysis. NIRS: near-infrared spectroscopy; MAP: mean arterial pressure; PRC: packed red cells; ICP: intracranial pressure.

### 2.3 Intervention protocol

Patients in the intervention group received continuous perioperative monitoring of rSO_2_ using a NIRS device (Root® monitoring platform, Masimo Corporation, Irvine, CA, USA). Baseline rSO_2_ values were recorded after sensors had been in place for at least 1 min and stabilized. Data were recorded every 15 s throughout the perioperative period and for 10 days after ICU discharge.

A protocol-driven response was followed to maintain rSO_2_ at or above 55% of baseline: (1) verify patient head position and inspect for facial plethora; (2) adjust ventilator settings to maintain PaCO_2_ ≥40 mmHg; (3) administer intravenous norepinephrine in 40 μg increments if mean arterial pressure (MAP) <65 mmHg; (4) increase inotropic support with dobutamine if CI <2.0 L/(min·m^2^); (5) increase FiO_2_ for ongoing desaturation; (6) transfuse packed red blood cells if hemoglobin <70 g/L (Fig. 1).

Patients in the control group received standard neurocritical care without rSO_2_ monitoring. All patients were managed in a closed, protocol-driven neuro-ICU to maintain hemoglobin levels (>70 g/L), blood glucose (4.4 – 10.0 mmol/L) and MAP (≥65 mmHg) (Fig. 1).^18^, ^19^, ^20^, ^21^ The care was provided exclusively by an intensivist-led team without direct influence from neurosurgeons or anesthesiologists.

### 2.4 Clinical management protocols

Sedation was titrated using a combination of fentanyl, propofol, midazolam, and cisatracurium. Sedation depth was assessed using the Richmond agitation-sedation scale, targeting a score of < −3, and the bispectral index, targeting 40 – 60. Ventilation was provided in volume-control mode, with tidal volume set to 8 mL/kg, respiratory rate adjusted to maintain normocapnia, FiO_2_ titrated to maintain SpO_2_> 95%, and inspiratory-to-expiratory ratio set at 1∶2.

### 2.5 Outcomes measured

The primary outcomes included hospital mortality, ICU length of stay (LoS), hospital LoS and incidence of major complications. The secondary outcomes included neurological recovery assessed using the modified Rankin scale (mRS, a 0 – 6 scale for degree of disability or dependence) and Glasgow outcome scale–extended (an 8-point scale for functional outcomes).

Cerebral desaturation was defined as rSO_2_ falling below 55% of baseline for 1 min or longer.^22^, ^23^, ^24^, ^25^ Both mean and minimum rSO_2_ values were recorded. To assess cerebral autoregulation, the Laser-Doppler index was computed by correlating cerebral perfusion pressure (CPP) with Laser-Doppler readings, and the cerebral oximetry index was calculated by correlating CPP with rSO_2_ measurements. For both indices, 30 data points from 15-s averaged intervals over 450 s were used per computation.

### 2.6 Ethical considerations

The study was approved by the Institutional Review Board of the Royal Thai Army Medical Department (Approval No. S069h/64, dated December 15, 2021) and registered at ClinicalTrials.gov (ID: NCT06306950). The study followed the Council for International Organizations of Medical Sciences 2012 guidelines and the International Conference on Harmonization – Good Clinical Practice principles (IRBRTA 1818/2564). All study procedures adhered strictly to applicable ethical standards and regulations.

### 2.7 Statistical analysis

Statistical analyses were performed using SPSS Version 26.0 (IBM Corp., Armonk, NY, USA). Categorical variables were summarized using frequency and percentage and compared using the Chi-square test or Fisher's exact test, as appropriate. Continuous variables were reported as mean ± standard deviation and analyzed using unpaired *t*-tests or ANOVA. Non-normally distributed variables were analyzed using the Wilcoxon rank-sum test. A *p* value <0.05 was considered statistically significant.

## 3 Results

### 3.1 Study population

As summarized in Table 1, the two groups were well-matched with respect to baseline demographic and clinical characteristics. The mean age was (63.2 ± 14.5) years in the NIRS group and (58.5 ± 14.2) years in the control group. No significant differences were identified between the two groups regarding injury severity (the acute physiology and chronic health evaluation II score, sequential organ failure assessment score and GCS), physiological variables (body mass index, MAP, intracranial pressure (ICP)), neurological status at admission (pupillary reactivity), pre-existing comorbidities, etc. (all *p* > 0.05). These findings confirm a balanced distribution of baseline risk factors across both groups, allowing for a valid comparison of outcomes.

**Table 1:** Baseline demographic and clinical characteristics of sTBI patients.

Variables	Intervention group (*n* = 40)	Control group (*n* = 40)	Statistic value	*p* value
Male	29 (72.5)	28 (70.0)	*χ*^2^ = 0.06	0.808
Age (years)	63.2 ± 14.5	58.5 ± 14.2	*t* = 1.46	0.148
Injury type			*χ*^2^ = 0.63	0.729
Blunt	36 (90.0)	34 (85.0)		
Penetrating	2 (5.0)	3 (7.5)		
Combined blunt and penetrating	2 (5.0)	3 (7.5)		
Injury mechanism			*χ*^2^ = 1.12	0.571
Accidental	38 (95.0)	36 (90.0)		
Self-inflicted	1 (2.5)	1 (2.5)		
Assault	1 (2.5)	3 (7.5)		
BMI (kg/m^2^)	22.23 ± 2.84	22.18 ± 2.91	*t* = 0.08	0.949
Underlying disease				
Hypertension	34 (85.0)	29 (72.5)	*χ*^2^ = 1.90	0.168
Dyslipidemia	24 (60.0)	20 (50.0)	*χ*^2^ = 0.80	0.371
Diabetes mellitus	28 (70.0)	27 (67.5)	*χ*^2^ = 0.06	0.806
Coronary artery disease	16 (40.0)	14 (35.0)	*χ*^2^ = 0.21	0.647
Previous cerebrovascular disease	7 (17.5)	5 (12.5)	*χ*^2^ = 0.39	0.532
SOFA score	6.7 ± 0.7	6.7 ± 1.1	*t* = 0.00	1.000
APACHE II score	13.4 ± 1.3	13.5 ± 1.5	*t* = −0.41	0.684
Hematocrit (%)	30.5 ± 6.2	30.6 ± 6.6	*t* = −0.08	0.936
Hemoglobin (g/L)	72.0 ± 17.2	72.3 ± 14.8	*t* = −0.08	0.936
Norepinephrine dose (μg/kg/min)	0.15 ± 0.07	0.12 ± 0.08	*t* = 1.79	0.077
ICP intracranial pressure (mmHg)	14.4 ± 1.3	14.8 ± 1.1	*t* = −1.47	0.145
Mean arterial pressure (mmHg)	65.0 ± 4.7	67.3 ± 6.5	*t* = −1.88	0.064
Cardiac index (L/min/m^2^)	3.4 ± 1.3	3.8 ± 1.1	*t* = −1.50	0.138
GCS at admission	5.4 ± 1.3	5.8 ± 1.1	*t* = −1.47	0.145
Surgical cases	34 (85.0)	29 (72.5)	χ^2^ = 1.90	0.168

Note: Data are presented as n(%) for categorical variables or mean ± standard deviation (SD) for continuous variables. The intervention group received continuous cerebral NIRS-guided monitoring of rSO_2_, whereas the control group received standard neurocritical care. No statistically significant differences were identified between groups at baseline, indicating well-balanced cohorts.

sTBI: severe traumatic brain injury; BMI: body mass index; SOFA: sequential organ failure assessment; APACHE-II: acute physiology and chronic health evaluation II; GCS: Glasgow coma scale; NIRS: near-infrared reflectance spectroscopy; rSO₂: regional cerebral oxygen saturation.

### 3.2 Clinical outcomes

The hospital mortality rate was slightly lower in the NIRS group (45%, 18/40) compared to the control group (47.5%, 19/40). Although the absolute difference was modest, it reached statistical significance (*p* = 0.043), suggesting a potential early survival benefit from NIRS-guided intervention, though clinical interpretation should be cautious given the exploratory nature of the trial.

At the 1-year follow-up, a significantly higher proportion of patients in the NIRS group achieved favorable neurological outcomes, defined as a Glasgow outcome scale–extended score ≥5, compared to the control group (25% *vs.* 5%, *p* = 0.006). This indicates a potential long-term benefit of the NIRS-guided management approach on neurological recovery. However, no statistically significant differences were observed between the two groups with respect to ICU and hospital LoS, and the number of ventilator-free or vasopressor-free days. These parameters suggest that while NIRS guidance may influence long-term neurological outcomes, it may not significantly impact the duration of acute critical care support.

### 3.3 Complication rates

As shown in Table 2, the incidence of major complications commonly associated with sTBI was similar between groups, including myocardial infarction, new-onset stroke, ventilator-associated pneumonia, etc. Among all complications, renal failure was the most frequently observed complication in both groups.

**Table 2:** Comparison of morbidity, mortality, and functional outcomes of sTBI patients between intervention and control groups.

Variable	Intervention group (*n* = 40)	Control group (*n* = 40)	*p* value
Complications, *n* (%) [95% *CI*]			
Myocardial infarction	3 (7.5) [1.6 – 20.4]	5 (12.5) [4.2 – 26.8]	0.438
New-onset stroke	3 (7.5) [1.6 – 20.4]	5 (12.5) [4.2 – 26.8]	0.438
Ventilator-associated pneumonia	6 (15.0) [5.7 – 29.8]	8 (20.0) [9.1 – 35.6]	0.532
Recurrent intracranial bleeding	2 (5.0) [0.6 – 16.9]	5 (12.5) [4.2 – 26.8]	0.218
Neurosurgical re-intervention	5 (12.5) [4.2 – 26.8]	6 (15.0) [5.7 – 29.8]	0.729
Renal failure	8 (20.0) [9.1 – 35.6]	12 (30.0) [16.6 – 46.5]	0.263
mRS at 1 year			
No. of cases with mRS being 1/2/3/4/5/6	2/4/4/1/1/18	0/0/2/4/5/19	0.043∗
Good outcomes (mRS 0 – 3)	10 (25.0)	2 (5.0)	0.006∗
Poor outcomes (mRS 4 – 6)	20 (50.0)	28 (70.0)	0.072
ICU length of stay (day)	10 (8 – 12)	10 (8 – 12)	1.000
Ventilator-free days	3 (2 – 4)	3 (2 – 4)	1.000
Vasopressor-free days	2 (1 – 3)	2 (1 – 3)	1.000

Note: The intervention group received continuous cerebral NIRS-guided monitoring of rSO_2_ and the control group received standard neurocritical care. Values are expressed as number (percentage) for categorical variables and median (interquartile range) for continuous variables. Incidence is presented with 95% *CI* where applicable. Comparisons between groups were made using the Chi-square test for categorical data and the Mann–Whitney *U* test for non-parametric continuous data.

CI: confidence interval; sTBI: severe traumatic brain injury; mRS: modified rankin scale; ICU: intensive care unit; NIRS: near-infrared reflectance spectroscopy.

In a multivariate analysis, two factors were identified as independent predictors of increased mortality: CI ≤2.0 L/(min·m^2^) (hazard ratio = 86.45, *p* = 0.021) and MAP ≤65 mmHg (hazard ratio = 8.23, *p* = 0.001). These results emphasize the critical importance of maintaining adequate hemodynamic parameters in patients with sTBI.

### 3.4 Correlation analysis

In the NIRS group, a statistically significant positive correlation was found between mean rSO_2_ levels and ICU LoS (*r* = 0.459, *p* < 0.001), as illustrated in Fig. 2. This correlation likely reflects the observation that patients requiring prolonged ICU care often received more sustained NIRS-guided interventions, which in turn may have contributed to improvements in cerebral oxygenation and recovery trajectories.

**Fig. 2:**
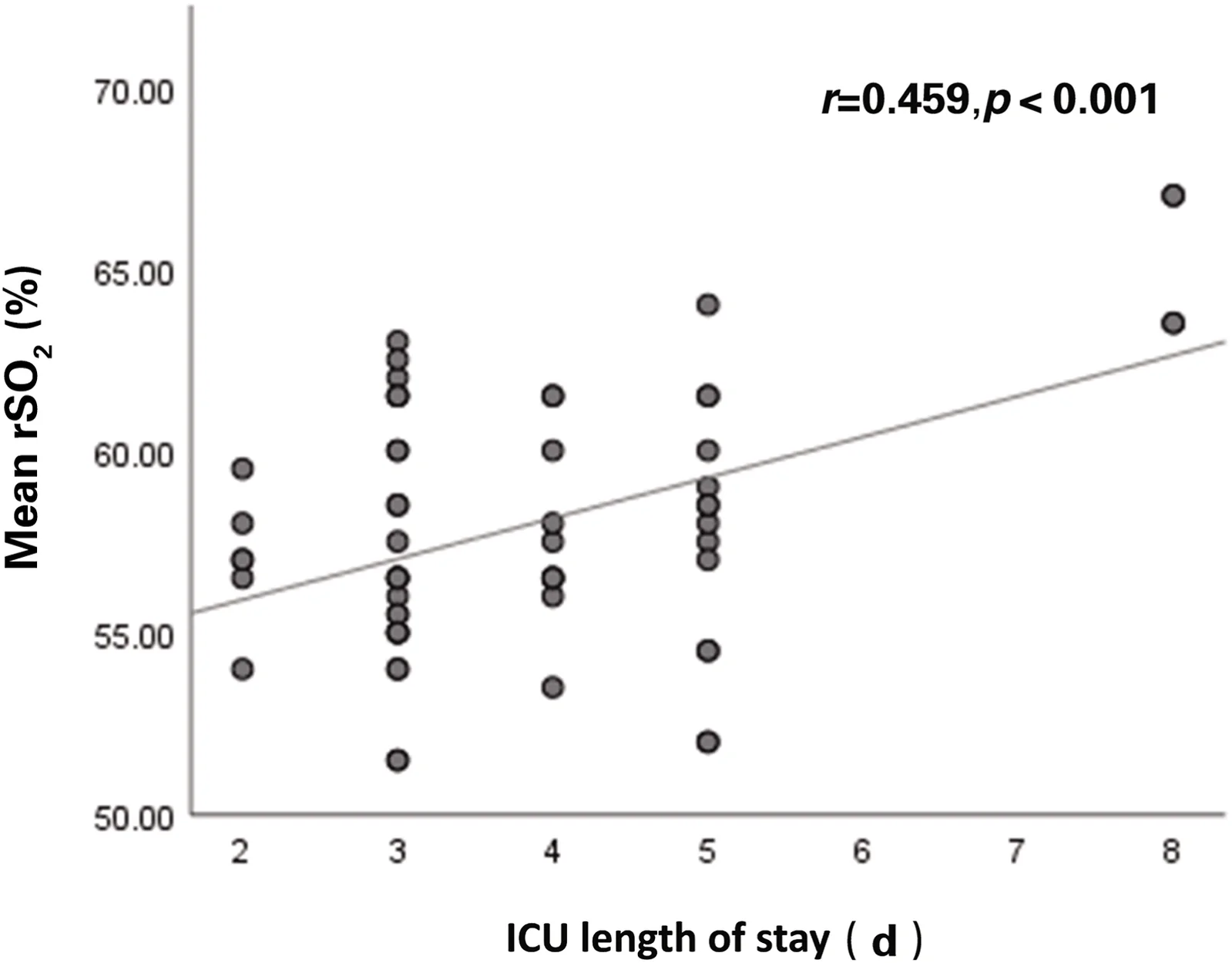
Pearson correlation demonstrates a statistically significant positive correlation between mean rSO_2_ values and ICU length of stay in patients managed with the NIRS protocol. A moderate correlation was observed (*r* = 0.459, *p* < 0.001), indicating that patients with prolonged ICU stays tend to maintain higher average rSO_2_ levels, likely due to ongoing, protocol-driven optimization of cerebral oxygenation. rSO₂: regional cerebral oxygen saturation. ;ICU: intensive care unit; d: day; NIRS: near-infrared reflectance spectroscopy.

In contrast, a weak and non-significant correlation was noted between mean rSO_2_ values and total hospital LoS (*r* = 0.169, *p* = 0.197, Fig. 3), suggesting that the effect of NIRS guidance was more pronounced in the ICU setting than in the overall hospitalization period.

**Fig. 3:**
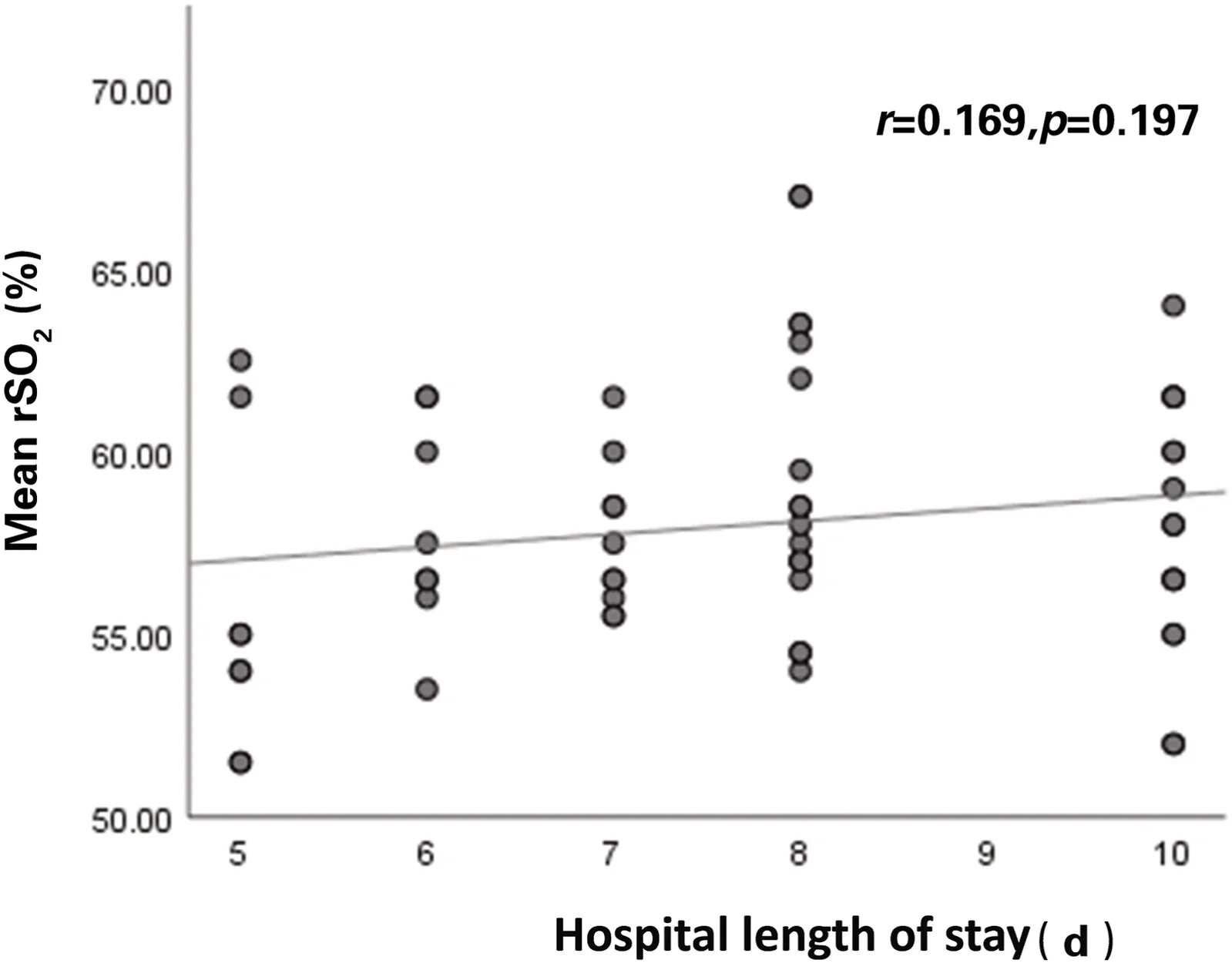
Pearson correlation illustrates the relationship between mean rSO_2_ values and total hospital length of stay among patients in the NIRS intervention group. Although a positive trend is observed (*r* = 0.169), the correlation did not reach statistical significance (*p* = 0.197), suggesting that mean rSO_2_ alone may not be a strong predictor of overall hospitalization duration. rSO₂: regional cerebral oxygen saturation; d: day; NIRS: near-infrared reflectance spectroscopy.

### 3.5 Cerebral autoregulation assessment

Both the cerebral oximetry index and Laser-Doppler index showed lower values when CPP was <50 mmHg, and higher values when CPP exceeded 80 mmHg, consistent with intact autoregulatory responses. This supports the role of NIRS not only in guiding interventions but also as a potential tool for assessing autoregulation in real time. Fig. 4 illustrates the utility of NIRS monitoring in detecting dynamic changes in cerebral perfusion status.

**Fig. 4:**
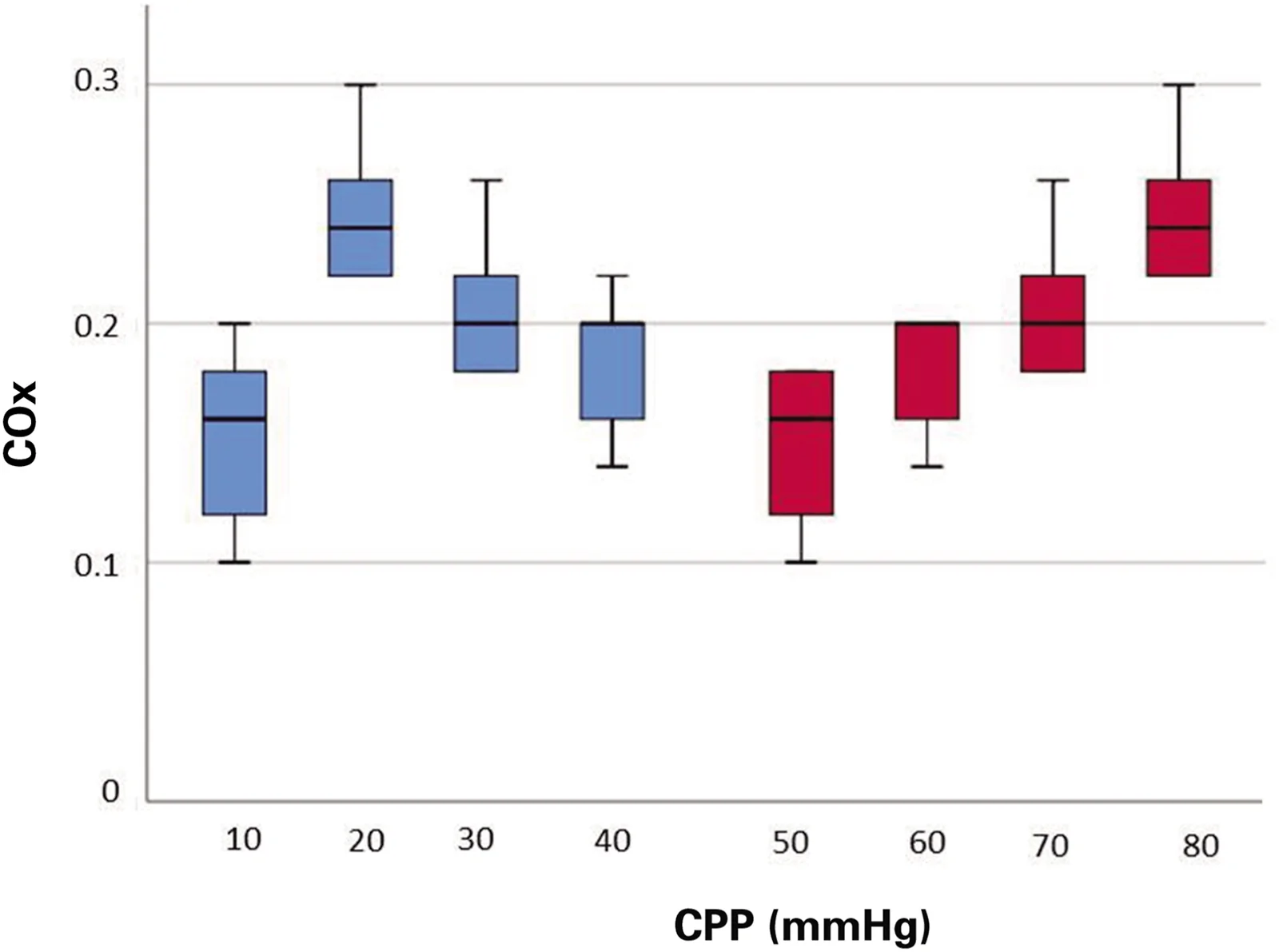
COx and autoregulation patterns relative to CPP. The COx values were concordant with expected physiological patterns, showing lower values (indicating preserved autoregulation) when CPP exceeded 50 mmHg, and higher values (indicating impaired autoregulation) when CPP dropped below 80 mmHg. These findings support the utility of NIRS in tracking cerebral autoregulatory status in patients with sTBI. Cox: cerebral oximetry index; CPP: cerebral perfusion pressure; NIRS: near-infrared reflectance spectroscopy; sTBI: severe traumatic brain injury.

## 4 Discussion

This prospective pilot study evaluated the feasibility and clinical impact of NIRS-guided cerebral oxygenation monitoring in patients with sTBI. Although the reduction in overall complication rates did not reach statistical significance, our findings are consistent with previous studies demonstrating that maintaining adequate cerebral oxygenation is associated with improved neurological outcomes and reduced organ dysfunction.^1^^,^^18^^,^^19^^,^^22^ These results support the growing body of evidence that NIRS-guided management may provide clinically meaningful benefits in high-risk populations.

While overall complication rates were similar between groups, patients in the intervention group demonstrated improved functional outcomes and a trend toward reduced organ dysfunction. These findings are consistent with prior studies showing that prevention of cerebral desaturation may attenuate secondary brain injury and improve recovery trajectories.^18^^,^^19^ Mechanistically, cerebral hypoxia contributes to neuronal injury through mitochondrial dysfunction, oxidative stress, and impaired autoregulation, all of which are key drivers of secondary injury in sTBI.^21^ These observations suggest that the application of NIRS-guided interventions may have mitigated secondary cerebral and systemic insults, resulting in better overall clinical trajectories.

A significant positive correlation between mean rSO_2_ and ICU LoS (*r* = 0.459) was observed in the intervention group. This finding likely reflects the fact that patients requiring prolonged ICU care underwent more intensive monitoring and sustained NIRS-guided optimization. Continuous cerebral oximetry enables early detection of hypoxic episodes and facilitates timely interventions, including optimization of MAP, CO, and arterial carbon dioxide levels, which collectively improve cerebral and systemic oxygen delivery.^20^, ^21^, ^22^, ^23^, ^24^, ^25^.

Additionally, the observed association between prolonged monitoring and improved rSO_2_ values supports previous findings that minimizing cerebral desaturation episodes is associated with improved outcomes following brain injury.^18^^,^^19^ NIRS serves as a dynamic surrogate marker of cerebral perfusion, allowing clinicians to detect early hemodynamic instability and intervene before irreversible injury occurs.^20^^,^^24^

The primary goal of the NIRS-guided protocol was to maintain cerebral oxygenation above a threshold (rSO_2_ ≥ 55%) by implementing timely physiological interventions. These included optimizing head position, correcting hypo- or hypercapnia, increasing MAP, enhancing CO, and, when necessary, transfusing red blood cells. In most cases, these interventions successfully restored rSO_2_ to target levels, with an overall success rate of 80%. Notably, there were no significant differences between groups in hemoglobin or hematocrit values, suggesting that cerebral oximetry did not result in overtreatment or increased transfusion rates.

The absence of additional risks and the integration of NIRS within standard clinical practice protocols support the safety and feasibility of this approach. These findings reinforce the concept of using the brain as an index organ, with cerebral oxygenation serving as a sensitive and early indicator of systemic and intracranial hemodynamic disturbances. In the context of sTBI, where cerebral autoregulation is often impaired and secondary injury is common, real-time cerebral monitoring can offer critical feedback for clinicians navigating complex therapeutic decisions.

Despite its advantages, cerebral oximetry must be interpreted within the broader context of neurophysiological instability. Variability in baseline rSO_2_ readings driven by ischemic insults, brain swelling, intracranial hypertension, and other secondary injuries complicates interpretation. Moreover, while NIRS is not currently used as a standalone modality for brain monitoring, it may offer valuable complementary insights when used alongside other monitoring tools such as ICP, brain tissue oxygen, and transcranial Doppler.

Notably, prior studies have supported the use of cerebral oximetry thresholds to detect critical desaturation events. Lam et al.^20^ demonstrated that a ≥13% reduction in the tissue oxygenation index from baseline could indicate clinically significant cerebral hypoxia. The present study's findings align with this literature, supporting the clinical utility of early detection and intervention using NIRS technology.

NIRS offers several practical advantages: it is portable, non-invasive, and relatively low-cost, making it ideal for bedside use in emergency departments, operating rooms, and ICUs. Furthermore, it enables dynamic monitoring of neuro-hemodynamics, including: regional cerebral oxygenation, assessment of autoregulation via cerebral oximetry index, and monitoring of regional cerebral blood flow using Doppler signals.

Clinically, NIRS may aid in calculating the optimal MAP for individual autoregulation, predicting risk of cerebral hyperperfusion syndrome, and identifying early signs of intracranial hypertension or seizures, which are often accompanied by abrupt changes in rSO_2_. Its application may even extend to prehospital care, where it can help detect expanding intracranial hematomas and guide early resuscitation and triage decisions during transport to specialized trauma centers.

Despite its advantages, NIRS has several limitations. Measurements may be influenced by extracranial tissue perfusion, potentially reducing accuracy. In addition, the limited depth of light penetration restricts monitoring primarily to cortical regions, which may not reflect deeper brain structures. There is also considerable inter-individual variability in baseline rSO_2_ values, making trend-based interpretation more reliable than absolute thresholds. Furthermore, NIRS does not directly measure cerebral blood flow or metabolism and should therefore be used as an adjunct to other neuromonitoring modalities, such as ICP and brain tissue oxygen monitoring.^21^ This study has several limitations. As a pilot trial, the sample size was relatively small, limiting the power to detect subtle differences in secondary outcomes. The exploratory nature of the study means that findings should be interpreted cautiously and confirmed through larger, multicenter trials. Moreover, while blinding was maintained for outcome assessors, the ICU team was unblinded to rSO_2_ values, which could introduce performance bias. Additionally, although the interventions were standardized and protocol-driven, there may have been variability in clinical decision-making. This pilot study suggests that NIRS-guided cerebral oxygenation monitoring is a feasible and safe strategy with potential clinical benefits in patients with sTBI. By enabling early detection of cerebral hypoxia and facilitating targeted physiological interventions, NIRS may help mitigate secondary brain injury and improve long-term outcomes. However, larger multicenter randomized trials are required to confirm these findings and to establish standardized clinical protocols.

In conclusion, NIRS can be an invaluable tool for guiding interventions and monitoring patients with sTBI to prevent cerebral deoxygenation. Continuous monitoring of cerebral rSO_2_ in these patients, coupled with efforts to prevent profound cerebral deoxygenation, was associated with improved neurological outcomes at 1-year follow-up. While the observed reduction in mortality was modest and not clinically significant, there was a statistically significant improvement in functional outcomes (modified Rankin scale 0 – 3). Factors such as a CI < 2 L/(min·m^2^) and MAP ≤65 mmHg were identified as key predictors of hospital mortality.

## CRediT authorship contribution statement

**Panu Boontoterm:** Writing – review & editing, Writing – original draft, Visualization, Validation, Supervision, Software, Resources, Project administration, Methodology, Investigation, Funding acquisition, Formal analysis, Data curation, Conceptualization. **Siraruj Sakoolnamarka:** Visualization, Validation, Supervision, Software, Resources, Project administration, Methodology, Investigation, Funding acquisition, Formal analysis, Data curation, Conceptualization. **Karanarak Urasyanandana:** Writing – original draft, Visualization, Validation, Supervision, Software, Resources, Project administration, Methodology, Investigation, Funding acquisition, Formal analysis, Data curation, Conceptualization. **Pusit Fuengfoo:** Writing – review & editing, Visualization, Validation, Supervision, Software, Resources, Project administration, Methodology, Investigation, Funding acquisition, Formal analysis, Data curation, Conceptualization.

## Ethical statement

The collected data have been decrypted and received ethical approval.

## Funding

Nil.

## Declaration of competing interest

The authors declare that the research was conducted in the absence of any commercial or financial relationships that could be construed as a potential conflict of interest.
